# The Effect of Contextual Variables on Match Performance across Different Playing Positions in Professional Portuguese Soccer Players

**DOI:** 10.3390/ijerph18105175

**Published:** 2021-05-13

**Authors:** Joel Barrera, Hugo Sarmento, Filipe Manuel Clemente, Adam Field, António J. Figueiredo

**Affiliations:** 1 Faculty of Sport Sciences and Physical Education, University of Coimbra, 3000-248 Coimbra, Portugal; afigueiredo@fcdef.uc.pt; 2 Research Unit for Sport and Physical Activity (CIDAF), 3000-248 Coimbra, Portugal; 3 Escola Superior Desporto e Lazer, Instituto Politécnico de Viana do Castelo, Rua Escola Industrial e Comercial de Nun’Álvares, 4900-347 Viana do Castelo, Portugal; filipe.clemente5@gmail.com; 4 Instituto de Telecomunicações, Delegação da Covilhã, 1049-001 Lisboa, Portugal; 5 School of Human and Health Sciences, University of Huddersfield, Huddersfield HD1 3DH, UK; Adam.field@hud.ac.uk

**Keywords:** football, monitoring, performance, professional team

## Abstract

This study investigated the position-specific physical demands of professional Portuguese players. The effects of situational variables on the physical performance demands were also analysed (match location, match half and match result). Match performance observations were collected using Global Navigation Satellite System devices across 11 matches during a competitive season (2019–2020). Data were analysed according to five playing positions: goalkeepers (*n* = 11), central defenders (*n* = 42), wide defenders (*n* = 31), central midfielders (*n* = 34), open attackers (*n* = 28), and centre forwards (*n* = 14). Central midfield players completed the greatest total distance (10,787 ± 1536 m), while central defenders covered the least distance (9272 ± 455; *p* < 0.001). Open attackers covered the greatest high and very-high-speed distance (1504 ± 363 m), number of high-speed decelerations per match (11 ± 4) and were the fastest players (30.6 ± 1.5 km/h), along with center forwards (30.6 ± 2.0 km/h), versus all other positions (*p* < 0.05). Greater distances were performed in teams that were winning (9978 ± 1963 m) or drawing (10,395 ± 875 m) versus losing (9415 ± 2050) *p* = 0.036 and *p* = 0.006, respectively. Increases in distance covered at walking speeds were observed during the 2nd half (1574 ± 179 m) compared with the 1st half (1483 ± 176; (*p* < 0.003). A higher number of decelerations across all speeds were performed in the 1st half (144 ± 39) versus the 2nd half (135 ± 37). The distance covered in home matches (10,206 ± 1926 m) far exceeded away matches (9471 ± 1932 m; *p* < 0.001). The number of faster accelerations were higher in away (7 ± 5) versus home matches (6 ± 4; *p* < 0.049). The data demonstrate the different physical demands of each playing position and suggest that situational variables influence physical performance. These findings suggest position-specific physical training is required to condition players for the bespoke demands of each playing position.

## 1. Introduction

The physical performance demands of soccer match-play have been researched extensively in English Premier League soccer players [1,2,3,4], and Australian, Italian [5] and Spanish league players [6]. Classic and contemporary match observations suggest professional soccer players cover between 9 and 14 km during match-play [7,8,9]. Studies primarily of English players also indicate that during competitive encounters, elite soccer players cover between 0.7–3.9 km high-speed distance [8,10] and 0.2–0.6 km sprint distance [11,12], while Spanish players in friendly matches cover smaller distances in these same variables [13]. English players are also reported to perform ~656 accelerations and ~612 decelerations during match-play [14], and Spanish players ~581 accelerations [13]. It is estimated that players spend the majority (80–90%) of matches performing low and medium intensity activities, whereas the decisive moments of a match largely require high-speed and sprint actions [15,16]. However, whilst the match demands have been clearly outlined in professional English players, there remain few match-play studies evaluating the demands of professional Portuguese players. Therefore, elucidating the match demands of professional Portuguese players appears warranted.

During competitive English Premier League matches, there appear to be large differences in the physical running output of different playing positions [17]. The greatest distance seems to be covered by midfield players (11.5 km) [15,18], whilst forwards and defenders cover lesser distances during match-play (10–10.5 km) [15]. A major limitation of the studies discriminating between playing positions is that small samples are used; thus, findings are often difficult to accurately differentiate across varying positional roles. Therefore, while the running demands during matches appear to differ, there are few large-scale studies of professional players elucidating the match demands between different positional roles. Assessing the difference between positional demands may help tailor individual training processes towards improving training drills for the needs of individual playing positions.

Given that soccer is associated with many complex logistical factors, it is plausible that situational variables influence match running profiles. It has also been identified that performance metrics can differ according to specific match factors [19,20]. One such contextual variable shown to influence physical performance is match half, with evidence suggesting that greater running distance is covered during the 1st half versus the 2nd half of matches [21,22]. Match location impacts technical performance [23], although few large-scale studies have assessed whether competing in home or away matches can influence the physical running metrics of soccer players. Another factor that has been investigated for its impact on performance is match status (Lago, 2009), which refers to whether a team wins, draws or loses a competitive match. Spanish La Liga soccer players are reported to cover greater distances when losing versus matches that are being drawn or won [24]. These findings are consistent with a separate study also evaluating the running profiles of Spanish La Liga players, which demonstrates that lesser high-speed running is performed when winning versus losing matches (Lago et al., 2010). Further research is required that develops an understanding of the influence of situational variables on soccer-specific running metrics across different leagues and countries. Considering the different cultures across European countries, it is logical to assume that the contextual variables influencing performance in some leagues may not apply to other countries. Therefore, assessing the influence that situational variables have on physical performance in professional Portuguese players will provide novel interpretations.

The aim of this study was to evaluate the physical performance of professional Portuguese soccer players across different positional roles. The interactive effects between match half, location, and status on physical performance metrics across official Portuguese matches were also assessed.

## 2. Materials and Methods

### 2.1. Experimental Approach to the Problem

A quasi-experimental design was used to evaluate the activity profile of different positional roles in competitive professional soccer matches. Those who played the entire 90 min (including added time) during the 2019–2020 (August to May) season were included for analyses. The matches were played between 11:00 am and 20:30 pm, and all players refrained from strenuous activity for 24 h prior to each match. All matches were played on natural turf. Given the observational nature of the research, no intervention or attempts were made to influence players performance, nor was any feedback provided to players throughout the entirety of data collection. 

### 2.2. Participants 

A total of 25 professional soccer players (age: 24.9 ± 4.2 years, stature: 180.9 ± 6.3 cm and body mass: 78.5 ± 8.5 kg) were monitored over 11 official matches in the Portuguese LigaPro (second tier). The players were classified into six different positions: goalkeepers (GK), wide defenders (WD), central defenders (CD), center midfielder (CM), open attackers (OA) and center forward (CF). The athletes who did not complete the entire duration of all 11 matches were excluded from analyses (*n* = 38). Each player provided informed consent, as did the leadership of the Coimbra Academic Association club. This study was approved by the scientific council and the ethics committee of the University of Coimbra (CE/FCDEF-UC/00692021). At the beginning of the season each player was evaluated by the club’s medical staff and underwent the Portuguese Football Federation’s mandatory medical examinations.

### 2.3. Experimental Procedure

Stature was determined using a SECA wall stadiometer (range 10–230 cm, division 1 mm). Body mass was determined through a multifrequency bioimpedance device (InBody770). Players voided their bowels and bladder before anthropometric characteristics were taken [25]. Assessments were conducted ≥3 h after the last meal with each player consuming approximately 500 mL of water ~2 h before evaluation to standardise hydration. Matches were analysed both across halves (1st and 2nd) and 15-min segments (0–15, 16–30, 31–45, 46–60, 61–75, 76–90 min). Additional (‘injury’) time for the first and second halves were also separated for analyses. The data were filtered to exclude any activity before kickoff (warm-up), during and reactivation after half-time, and cool-down at the end of the match.

Match data were recorded using portable GNSS (SPI HPU, GPSports, Canberra, Australia) at a sampling frequency of 15 Hz. These devices have reported good inter-unit reliability (coefficient of variation [CV]: 1.9% for TD, 7.6% for speeds of 3.8–5.5 m/s/s and 12.1% for speeds ≥ 5.5 m/s/s). Previous models of the 15 Hz units have revealed a degree of error for TD (1.1%) and maximum speed running (<1%) [26,27]. At the end of each match, the data from the GNSS devices were extracted using the manufacturer’s software (Team AMS, Canberra, Australia). Match activities were categorised as the following: standing, very low speed walking (0–5.9 km·h^−1^), low speed walking (6–11.9 km·h^−1^), low speed jogging (12–13.9 km·h^−1^), medium speed running (14–17.9 km·h^−1^), high speed running (18–23.9 km·h^−1^) and sprinting (24 km·h^−1^). The different speed thresholds form the 11 official matches are summarised in Table 1.

### 2.4. Statistical Analysis 

Data were not normally distributed, and thus, non-parametric tests were used for analyses. Kruskal–Wallis was used to compare performance between the different positions and according to the match result. A Mann–Whitney U was performed for comparisons of match location and match half. Data were analysed using Statistical Package for the Social Sciences (IBM SPSS Statistics 24 for Windows; SPSS Inc., Chicago, IL, USA). Significance was accepted at *p* ≤ 0.05 prior to analyses.

## 3. Results

Detailed results according to playing position are presented in Table 2 and between-group comparisons in Table 3. The lowest TD was performed by CD, while the highest TD was covered by CM (10.787 ± 1.536 m; *p* < 0.001). The greatest high-speed distance in the sum of ZD4, 5 and 6 was performed by OA versus all other positions (*p* < 0.001). The WD, MC and OA demonstrated significantly higher values for the sum of accelerations and decelerations compared with CD and CF (*p* < 0.001).

It was identified that TD was higher for the tied and won matches (10.395 ± 87 m, and 9.978 ± 1.962 m, respectively) versus lost matches (9.414 ± 2.050 m). Significant differences were found for TD between drawing and losing (*p* = 0.036), and winning and losing (*p* = 0.006) (Table 4).

No significant differences were observed for average TD covered between the 1st (4942 ± 945 m) and 2nd (4868 ± 980) halves (*p* = 0.432). A decrease in the total number of decelerations in the 2nd half versus the 1st half was also identified (*p* = 0.036) (Table 5). Greater TD (*p* = 0.0002), TDP (*p* = 0.0008) and total displacements at low intensity (*p* = 0.00003) were observed in the home games in versus away matches. A higher number of Acc3 (*p* = 0.049) was observed in the away matches. (Table 6).

The results revealed that the highest number of sprints were performed by OA (*n* = 5.39 ± 2.6), while the CD (*n* = 2.47 ± 1.5) showed the lowest values (*p* = 0.000). Furthermore, the highest number of sprints based in accelerations (SBA) were performed in Q1 (*n* = 5.04 ± 2.9), while the lowest frequencies were identified during Q4 (*n* = 3.77 ± 2.5) (Table 7).

## 4. Discussion

The aim of this study was to analyse performance in professional Portuguese soccer players across different playing positions and contextual variables. Different positions require different physical demands, which are influenced by the location of the match and the result. Overall, players covered a mean TD of 9839 ± 1929 m, with midfielders covering the greatest distance and central defenders performing the least TD. Wingers covered the most high-speed distance, number of decelerations (3.0–4.0 m/s/s) and were the fastest players, along with center forwards. Players performed greater distances when their team drew or won, and reductions for some running metrics were observed in the 2nd half of matches. The number of faster accelerations (3.0–4.0 m/s/s) was higher in away versus home games. These data may be considered by coaches and physical practitioners as a guide for the training prescription for their teams.

The TD covered was similar with values reported by studies conducted in soccer players across other European countries [1,28]. Specifically, the mean TD covered was similar in the present study for CM (10,788 ± 1537 m) and OA (10,641 ± 1537 1011 m), compared to the 2006–2007 (10,679 ± 956) and 2012–13 (10,881 ± 885 m) seasons in the English Premier League [1]. However, lower sprint distances across all playing positions were reported in the present study (≤199.6 ± 89.9 m) versus the English Premier League players (232 ± 114 (2006–2007 season) and 350 ± 139 m (2012–2013 season)). Therefore, this study presents novel evidence that soccer players competing in the second Portuguese league cover similar distances during competitive match-play with English Premier League players, but lesser sprint distances. The discrepancy in sprint distance may be reflective of the players across both studies competing in different countries (tactical modifications) and tiers (high-standard athletes). This is largely supported by evidence that suggests high-speed and sprint performance is superior in higher level soccer players during match-play [11]. These data indicate that practitioners may direct their focus on the development of sprint capacity to increase physical performance of lower division players during match-play, considering the specific demands of each position for the training prescription, optimizing the preparation process.

High-speed running performance is thought to be capable of discriminating between competitive levels and is a better indicator of physical performance versus TD alone [1]. However, a paucity of published information is available on the differences in high-speed running across playing positions in the Portuguese soccer leagues. In relation to high-speed running performance across different playing positions in the current investigation, it appears that CD perform considerably less high-speed activity versus all other positional roles (excluding the GK). This observation largely agrees with a previous study assessing English Championship and Premier League players [3]. The study found that English championship (540 ± 129 m) and Premier League players (482 ± 116 m) cover considerably less high-speed running distance compared with all other outfield positions, showing significant performance differences between positions. In relation to the WD and OA players, the results are consistent with players of wider positions in the English Premier League high-speed running values [29]. These results suggest that training programmes for soccer players must be individualised and specific to the demands of the playing position to enhance physical performance during match-play.

The number of accelerations and decelerations present a similar pattern as identified in previous studies, with a greater quantity of actions reported in the 1st half versus the 2nd half [30], but it differs in that the position that performs the greatest number of accelerations in the present study is the CM, while in preparation games in Spanish soccer it is the CD [13], context that can influence these returns. Concerning the playing roles, the highest number of Acc1 (1.0–1.9 m/s/s) was performed by CM, who presented substantially higher values versus all positions. With reference to Acc2 (2.0–2.9 m/s/s), the highest quantity of actions was performed by the OA, who had differences with all positions except the WD, who also presented differences with CD, CF, and CM. Pertaining to Acc3 (3.0–4.0 m/s/s), the OA and CF achieved the highest performance and were considerably different compared to other positions (CM, CD, and WD). This suggests that the quantity of accelerations performed at different speeds varies depending on the demands of the position. These data can, therefore, be used as a guide to inform acceleration-based training paradigms that incorporate the different acceleration loads of each playing position.

Decelerations are also an important component to consider in soccer, since players must frequently decelerate at high speeds, placing lower-limb musculature under a high eccentric loading demand [31]. The number of decelerations (1.0–4.0 m/s/s) performed by CM players was higher than CD, CF, and OA. These results may reflect the greater CM involvement in the game, both in offensive and defensive tasks. However, the OA players performed significantly more Dec3 (3.0–4.0 m/s/s) than other positions. This is possibly linked with the fact that OAs tend to run faster (30.6 ± 1.5) than other players, which necessitates a higher frequency of rapid decelerations. These demanding actions are associated with increased fatigue, which decreases locomotive efficiency at the end of each half in competitive football and thus increases the likelihood of injury incidence [32]. In this way, both accelerations and decelerations are another indicator that shows the variability of effort between the different positions; therefore, it is recommended that the functional specificity and movement drills incorporated within training programmes expose players to the quantity of accelerations and decelerations reported in the current data.

Lower performance was observed in the games played away from home, which may be influenced by tactical approaches and defensive strategies adopted to limit the opposition rather than providing an attacking threat [33]. There may also be additional logistical issues involved with travelling that limits players preparation and reduces overall performance output and recovery [34]. Thus, this specific aspect should be considered when scheduling the weekly planning and periodisation of training sessions [35]. Greater very-low intensity (DZ1) activity was performed in the 2nd half versus the 1st half. This may be a subconscious self-pacing approach to conserve energy during the second half, to avoid significant reductions in high-speed capacity [36] or avoid fatigue-induced injury during the latter stages of match-play [37]. It is also possible that the reductions in intensity during the 2nd half are linked to a fatigue-induced inability to perform the actions rather than pacing strategies employed to maintain energy for the final moments of a match [38]. It is integral that physical capacity is maintained for the entire 90 min as approximately 57% of goals are scored in the 2nd half [39,40]. Therefore, it is crucial that players are conditioned to be able to compete for an entire 90 min and that substitutions are utilised for the players incapable of performing the required running output across an entire match [41].

The SBA estimated in 6 blocks of 15 min showed a gradual decrease as the game progressed in the 1st half, while during the 2nd half the trend was the opposite and the lowest performance was presented at the beginning and increased during the game. This is perhaps associated with declines in body temperature that occur due to passive recovery in the changing rooms and a short and ineffective reactivation at the beginning of the 2nd half [42]. This information should be used to better manage reactivation during matches and to create recovery contexts during training sessions that reflect what happens in competition. This may allow optimal adaptation to passive recovery and reduce insufficient performance during the start of each half. Adequate half-time re-warm-up and passive heat maintenance strategies may also enhance performance in the initial stages of the 2nd half [43]. Thus, adherence to such protocols may gain soccer teams the competitive advantage during the early stages of the second half of matches.

## 5. Conclusions

The quantification of the demands of second-division Portuguese matches through GNSS devices presents novel perspectives. The current data suggest that the physical demands of each playing position are impacted by match location and result. The demands of each playing position are reported throughout the article, with midfield players covering the most distance and largely performing the greatest quantity of actions throughout match-play. Players completed greater distances when their teams had a positive result (i.e., won or draw), and running performance largely decreased in the 2nd half of matches, an observation that is consistent with the literature. Based on the current findings, training drills should be position-specific, where appropriate, to prepare players for the physical capacity and running output of the various positional roles during matches. Future studies are required to evaluate match running profiles across the major European Leagues (German Bundesliga, French Ligue 1 and the Spanish La Liga) to compare with the current data and the wealth of observations available on English Premier League players.

## Figures and Tables

**Table 1:** Speed thresholds for the reported match variables.

Variables	Abbreviation		Rank
Total distance	TD m		0–≤24 km/h
Total distance by parts	TDP m		0–≤24 km/h
Zone 1 distance	ZD1 m	standing. walking very low intensity	0–5.9 km/h
Zone 2 distance	ZD2 m	low intensity walking	6–11.9 km/h
Zone 3 distance	ZD3 m	jog at low intensity	12–13.9 km/h
Zone 4 distance	ZD4 m	running at medium intensity	14–17.9 km/h
Zone 5 distance	ZD5 m	running high speed	18–23.9 km/h
Zone 6 distance	ZD6 m	Sprint	≤24 km/h
Sum distances (1,2,3)	ƩD m (1,2,3)		0–13.9 km/h
Sum distances (4,5,6)	ƩD m (4,5,6)		14–≤24 km/h
Acceleration 1	Acc1 m/s/s		1.0–1.9 m/s/s
Acceleration 2	Acc2 m/s/s		2.0–2.9 m/s/s
Acceleration 3	Acc3 m/s/s		3.0–4.0 m/s/s
Sum Accelerations (1,2,3)	ƩAcc (1,2,3) m/s/s		1.0–4.0 m/s/s
Deceleration 1	Des1 m/s/s		1.0–1.9 m/s/s
Deceleration 2	Des2 m/s/s		2.0–2.9 m/s/s
Deceleration 3	Des3 m/s/s		3.0–4.0 m/s/s
Sum Deceleration (1,2,3)	ƩDes (1,2,3) m/s/s		1.0–4.0 m/s/s
Maximum speed	MS km/h		≤24 km/h

**Table 2:** Distances covered across each speed threshold according to each playing position (Data are reported as mean ± SD).

Variables	GK *n* = 11			WD *n* = 31			CD *n* = 42			CM *n* = 34			OA *n* = 28			CF *n* = 14			Differences Between Positions	
	Mean		SD	Mean		SD	Mean		SD	Mean		SD	Mean		SD	Mean		SD	H de Kruskal-Wallis	Sig
TD m	4237.9	±	2342.1	10,582.4	±	504.4	9272.5	±	455.7	10,787.9	±	1536.8	10,640.6	±	1011.4	9336.9	±	673.6	90.451	0.0001
TDP m	2139.2	±	1243.9	5284.0	±	292.7	4636.2	±	263.9	5544.5	±	366.8	5320.3	±	535.8	4668.4	±	439.8	89.757	0.0001
ZD1 m	1320.5	±	139.8	1401.8	±	96.2	1672.0	±	129.1	1382.4	±	122.6	1679.6	±	98.6	1596.8	±	107.2	102.348	0.0001
ZD2 m	533.0	±	581.7	2010.8	±	149.3	1806.1	±	164.4	2175.2	±	209.0	1605.3	±	163.4	1764.3	±	206.4	95.502	0.0001
ZD3 m	92.9	±	158.9	533.6	±	77.8	433.8	±	84.0	692.0	±	144.0	529.5	±	100.7	375.2	±	63.7	92.521	0.0001
ZD4 m	126.4	±	260.6	707.3	±	115.2	475.6	±	102.7	853.9	±	194.3	741.8	±	172.2	479.4	±	56.9	94.934	0.0001
ZD5 m	55.7	±	162.4	472.2	±	110.0	211.8	±	58.3	380.9	±	95.1	561.4	±	143.2	338.4	±	71.2	107.543	0.0001
ZD6 m	8.5	±	28.3	155.1	±	76.5	33.9	±	23.9	56.9	±	41.1	199.6	±	89.9	110.9	±	64.9	102.121	0.0001
ƩD m (1,2,3)	1947.8	±	800.4	3947.8	±	222.4	3913.4	±	217.2	4251.2	±	246.7	3816.1	±	248.0	3737.8	±	310.3	63.108	0.0001
ƩD m (4,5,6)	191.4	±	451.0	1336.2	±	227.7	722.8	±	149.1	1293.3	±	251.1	1504.3	±	362.8	930.5	±	161.5	105.854	0.0001
Acc1 m/s/s	34.5	±	35.0	117.5	±	19.4	107.2	±	14.8	135.0	±	23.4	103.6	±	16.4	87.1	±	12.1	71.114	0.0001
Acc2 m/s/s	9.6	±	10.6	43.1	±	8.1	26.8	±	6.9	36.8	±	12.5	48.3	±	13.0	33.2	±	6.8	76.501	0.0001
Acc3 m/s/s	1.7	±	1.6	8.8	±	3.9	3.3	±	2.1	4.9	±	2.7	11.3	±	3.7	10.8	±	4.7	92.085	0.0001
ƩAcc (1,2,3) m/s/s	45.8	±	46.6	169.3	±	23.0	137.4	±	16.9	176.7	±	33.7	163.3	±	28.1	131.1	±	16.3	69.553	0.0001
Des1 m/s/s	31.7	±	28.0	99.3	±	15.5	95.5	±	12.7	116.1	±	17.8	83.1	±	12.5	74.9	±	10.7	81.279	0.0001
Des2 m/s/s	8.2	±	9.1	36.5	±	7.5	26.4	±	5.8	38.9	±	11.5	39.4	±	9.0	28.0	±	6.3	69.645	0.0001
Des3 m/s/s	2.5	±	4.6	17.9	±	5.2	10.4	±	4.2	12.9	±	5.2	25.6	±	7.7	19.8	±	4.1	90.250	0.0001
ƩDes (1,2,3) m/s/s	42.4	±	41.0	153.7	±	15.6	132.2	±	15.8	167.9	±	30.3	148.1	±	23.7	122.7	±	15.7	68.209	0.0001
MS km/h	20.2	±	3.1	29.7	±	1.9	27.4	±	2.2	28.0	±	2.1	30.6	±	1.5	30.6	±	2.0	70.830	0.0001

Note. Goalkeeper = GK Wide defenders = WD, central defenders = CD, center midfielder = CM, open attackers = OA) and center forward = CF.

**Table 3:** Comparisons between playing positions for each of the speed thresholds.

Variables	WD >	CD >	CM >	OA >	CF >
TD m	DC **/CD **		DL **/DC **/CD **	DC **/CD **	
TDP m	DC **/CD **		DL **/DC **/CD **	DC **/CD **	
ZD1 m		DL **/MC **		DL **/MC **/CD **	DL **
ZD2 m	DC **/EXT **/CD **	EXT **	DL **/DC **/EXT **/CD **		EXT **
ZD3 m	DC **/CD **	CD *	DL **/DC **/EXT **/CD **	DC **/CD **	
ZD4 m	DC **/CD **		DL **/DC **/CD **	DC **/CD **	
ZD5 m	DC **/MC **/CD **		DC **	DL **/DC **/MC **/CD **	DC **
ZD6 m	DC **/MC **/CD **		DC **	DL */DC **/MC **/CD **	DC **/MC **
ƩD m (1,2,3)	EXT */CD **		DL **/DC **/EXT **/CD **		
ƩD m (4,5,6)	DC **/CD **		DC **/CD **	DL **/DC **/MC **/CD **	DC **
Acc1 m/s/s	DC */EXT **/CD **	CD **	DL **/DC **/EXT **/CD **	CD **	
Acc2 m/s/s	DC **/MC */CD **		DC **	DC **/MC **/CD **	DC **
Acc3 m/s/s	DC **/MC **		DC **	DL */DC **/MC **	DC **/MC **
ƩAcc (1,2,3) m/s/s	DC **/CD **		DC **/CD **	DC **/CD **	
Des1 m/s/s	EXT ** /CD **	EXT **/CD **	DL **/DC **/EXT **/CD **	CD *	
Des2 m/s/s	DC **/CD **		DC **/CD **	DC **/CD **	
Des3 m/s/s	DC **/MC **		DC *	DL**/DC **/MC **/CD **	DC **/MC **
ƩDes (1,2,3) m/s/s	DC **/CD **		DC **/EXT */CD **	DC **/CD **	
MS km/h	DC **/MC **			DL **/DC **/MC **	DC **/MC **

Note. GK comparisons are omitted from analyses. Wide defenders = WD, central defenders = CD, center midfielder = CM, open attackers = OA) and center forward = CF. * *p* ≤ 0.05; ** *p* ≤ 0.001

**Table 4:** Distances covered across each speed threshold when the teams drew, lost, or won (Data reported as mean ± SD).

Variables	Draw *n* = 14			Lose *n* = 76			Win *n* = 70			Difference between Groups		Difference between Pairs		
	Mean		SD	Mean		SD	Mean		SD	H de Kruskal-Wallis	Sig	D >	L >	W >
TD m	10,395.1	±	875.0	9414.9	±	2050.2	9978.5	±	1962.5	9.342	0.009	*p* *		*p* **
TDP m	5197.6	±	452.4	4707.5	±	1048.2	5062.4	±	899.2	9.170	0.010			*p* **
ZD1 m	1518.4	±	186.3	1496.6	±	185.7	1565.6	±	175.2	3.996	0.136			*p* *
ZD2 m	1905.3	±	270.2	1706.6	±	489.2	1875.5	±	428.5	10.052	0.007			*p* **
ZD3 m	533.1	±	95.8	475.1	±	196.8	511.7	±	177.6	3.518	0.172			
ZD4 m	675.3	±	183.1	597.3	±	251.9	642.3	±	259.0	1.361	0.506			
ZD5 m	433.5	±	165.0	339.4	±	183.0	367.0	±	178.3	3.209	0.201			
ZD6 m	128.9	±	104.7	89.4	±	83.8	97.1	±	89.9	2.443	0.295			
ƩD m (1,2,3)	3958.3	±	179.6	3679.8	±	680.1	3954.4	±	570.5	20.241	0.000			*p* **
ƩD m (4,5,6)	1239.2	±	423.4	1027.6	±	458.0	1108.0	±	462.7	2.312	0.315			
Acc1 m/s/s	116.6	±	22.2	103.0	±	34.0	111.1	±	28.7	4.960	0.084			*p* *
Acc2 m/s/s	36.9	±	11.2	34.6	±	13.7	35.6	±	15.4	0.151	0.927			
Acc3 m/s/s	7.9	±	4.9	6.6	±	4.8	6.4	±	4.4	1.019	0.601			
ƩAcc (1,2,3) m/s/s	161.4	±	27.2	144.2	±	45.1	153.1	±	41.0	2.851	0.240			
Des1 m/s/s	94.2	±	17.4	89.3	±	29.0	95.0	±	23.4	2.407	0.300			
Des2 m/s/s	33.7	±	6.8	31.3	±	12.2	32.9	±	12.4	0.665	0.717			
Des3 m/s/s	18.1	±	7.9	14.6	±	8.1	15.5	±	8.6	2.141	0.343			
ƩDes (1,2,3) m/s/s	146.0	±	21.5	135.2	±	42.2	143.4	±	35.3	2.586	0.274			
MS km/h	29.8	±	1.8	28.1	±	3.4	28.3	±	3.2	3.944	0.139	*p* *		

* *p* ≤ 0.05; ** *p* ≤ 0.001.

**Table 5:** Distances covered across each speed threshold according to first and second half (data are reported as mean ± SD).

Variables	1st Part *n* = 80			2nd Part *n* = 80			Difference between 1st and 2nd part			
	Mean		SD	Mean		SD	U de Mann-Whitney	Sig	1st >	2nd >
TDP m	4942.7	±	945.2	4868.6	±	980.5	2969.5	0.432		
ZD1 m	1483.3	±	176.6	1574.1	±	179.3	2320.5	0.003		1st *
ZD2 m	1829.3	±	437.2	1766.5	±	471.0	2814.5	0.188		
ZD3 m	500.6	±	183.9	491.8	±	181.2	3124.0	0.795		
ZD4 m	639.9	±	254.0	607.7	±	246.5	2950.0	0.394		
ZD5 m	380.1	±	186.1	339.3	±	173.2	2776.0	0.148		
ZD6 m	106.5	±	91.9	86.0	±	84.3	2785.5	0.157		
ƩD m (1,2,3)	3814.7	±	585.8	3834.0	±	650.9	2987.0	0.467		
ƩD m (4,5,6)	1128.0	±	471.1	1034.6	±	444.0	2777.0	0.149		
Acc1 m/s/s	110.1	±	31.8	105.4	±	30.4	2819.0	0.193		
Acc2 m/s/s	36.1	±	15.2	34.4	±	13.3	3039.5	0.584		
Acc3 m/s/s	7.0	±	5.1	6.3	±	4.2	3072.5	0.663		
ƩAcc (1,2,3) m/s/s	153.1	±	43.1	146.1	±	41.2	2742.0	0.118		
Des1 m/s/s	94.5	±	26.5	90.0	±	25.1	2892.0	0.293		
Des2 m/s/s	33.6	±	12.5	30.7	±	11.2	2721.0	0.102		
Des3 m/s/s	16.3	±	9.0	14.3	±	7.5	2803.0	0.175		
ƩDes (1,2,3) m/s/s	144.4	±	38.7	135.1	±	36.7	2585.5	0.036	2nd *	
MS km/h	28.5	±	3.6	28.1	±	2.9	2761.5	0.134		

* *p* ≤ 0.05

**Table 6:** Distances covered across each speed threshold when the teams competed home or away (Data reported as mean ± SD).

Variables	Local *n* = 60			Visit *n* = 100			Difference between Groups			
	Mean		SD	Mean		SD	U de Mann-Whitney	Sig	L >	V >
TD m	10,208.0	±	1926.2	9470.8	±	1932.4	1948.0	0.0002	V **	
TDP m	5104.0	±	974.1	4786.6	±	937.4	2050.5	0.0008	V **	
ZD1 m	1549.8	±	190.3	1516.1	±	178.5	2719.0	0.3220		
ZD2 m	1892.5	±	476.4	1741.1	±	432.6	2109.5	0.0017	V **	
ZD3 m	530.5	±	193.3	475.6	±	172.7	2315.0	0.0158	V **	
ZD4 m	654.9	±	266.3	605.1	±	239.2	2648.0	0.2147		
ZD5 m	370.5	±	189.4	353.2	±	175.4	2866.0	0.6367		
ZD6 m	102.6	±	97.5	92.4	±	82.9	2896.0	0.7138		
ƩD m (1,2,3)	3974.3	±	625.1	3734.3	±	597.8	1666.0	0.000003	V **	
ƩD m (4,5,6)	1129.7	±	483.0	1052.3	±	443.4	2691.0	0.2761		
Acc1 m/s/s	111.7	±	32.1	105.3	±	30.4	2518.5	0.0896		
Acc2 m/s/s	34.4	±	15.5	35.7	±	13.5	2806.0	0.4940		
Acc3 m/s/s	5.6	±	4.0	7.3	±	4.9	2444.0	0.0493		L *
ƩAcc (1,2,3) m/s/s	151.8	±	44.4	148.3	±	41.0	2826.5	0.5408		
Des1 m/s/s	96.0	±	26.2	90.0	±	25.4	2464.0	0.0588		
Des2 m/s/s	34.1	±	12.9	31.0	±	11.2	2596.5	0.1547		
Des3 m/s/s	14.2	±	8.4	16.0	±	8.2	2603.5	0.1618		
ƩDes (1,2,3) m/s/s	144.3	±	39.4	137.0	±	36.9	2497.0	0.0762		
MS km/h	28.2	±	3.2	28.4	±	3.3	2906.0	0.7404		

* *p* ≤ 0.05; ** *p* ≤ 0.001.

**Table 7:** Summary of each 15-min period of matches, playing positions, match result, first and second half and match location (Data reported as mean ± SD).

Variables		*n*	Mean		SD	H de Kruskal-Wallis	Sig	U de Mann-Whitney
Quartile	00-15 = Q1 >	378	5.04	±	2.9	82.234	0.000	Q4 **/Q5 */Q7 **/Q8 **
	16-30 = Q2 >	360	4.68	±	3.0			Q7 **/Q8 **
	31-45 = Q3 >	335	4.72	±	2.8			Q4 */Q7 **/Q8 **
	46-60 = Q4 >	264	3.77	±	2.5			Q7 **/Q8 **
	61-75 = Q5 >	287	3.99	±	2.5			Q7 **/Q8 **
	76-90 = Q6 >	319	4.09	±	2.2			Q7 **/Q8 **
	More of 45 = Q7 >	28	1.27	±	0.5			
	More of 90 = Q8 >	106	2.12	±	1.1			Q7 **
Positions	GK >	41	1.71	±	1.0	107.057	0.000	
	WD >	526	4.78	±	2.7			GK **/CD **
	CD >	301	2.47	±	1.5			GK **
	CM >	444	4.11	±	2.6			GK **/CD **/
	OA >	544	5.39	±	2.6			GK **/CD **/CM **/CF *
	CF >	221	4.42	±	2.8			GK **CD **
Result	D >	400	4.35	±	2.9	1.091	0.579	No difference
	L >	761	3.90	±	2.6			
	W >	916	4.02	±	2.6			
Parts	1st >	1104	4.52	±	2.9			27,322.0/0.001/2da **
	2nd >	973	3.59	±	2.3			
Place	Local	702	3.84	±	2.6			465,352.0/0.433/No Dif
	Visit	1375	4.14	±	2.7			

* *p* ≤ 0.05; ** *p* ≤ 0.001.
